# A Novel Single Virus Infection System Reveals That Influenza Virus Preferentially Infects Cells in G1 Phase

**DOI:** 10.1371/journal.pone.0067011

**Published:** 2013-07-18

**Authors:** Ryuta Ueda, Tadao Sugiura, Shinichiro Kume, Akihiko Ichikawa, Steven Larsen, Hideaki Miyoshi, Hiroaki Hiramatsu, Yasuko Nagatsuka, Fumihito Arai, Yasuo Suzuki, Yoshio Hirabayashi, Toshio Fukuda, Ayae Honda

**Affiliations:** 1 Department of Frontier Bioscience, Hosei University, Koganei, Tokyo, Japan; 2 Department for Information Science, Nara Institute of Science and Technology, Ikoma, Nara, Japan; 3 Department of Micro-Nano Systems Engineering, Nagoya University, Nagoya, Aichi, Japan; 4 Department of Biomedical Science, Chubu University, Kasugai, Aichi, Japan; 5 Laboratory for Molecular Membrane Neuroscience, RIKEN, Wako, Saitama, Japan; 6 Department of Engineering, Nagoya University, Nagoya, Aichi, Japan; German Primate Center, Germany

## Abstract

**Background:**

Influenza virus attaches to sialic acid residues on the surface of host cells via the hemagglutinin (HA), a glycoprotein expressed on the viral envelope, and enters into the cytoplasm by receptor-mediated endocytosis. The viral genome is released and transported in to the nucleus, where transcription and replication take place. However, cellular factors affecting the influenza virus infection such as the cell cycle remain uncharacterized.

**Methods/Results:**

To resolve the influence of cell cycle on influenza virus infection, we performed a single-virus infection analysis using optical tweezers. Using this newly developed single-virus infection system, the fluorescence-labeled influenza virus was trapped on a microchip using a laser (1064 nm) at 0.6 W, transported, and released onto individual H292 human lung epithelial cells. Interestingly, the influenza virus attached selectively to cells in the G1-phase. To clarify the molecular differences between cells in G1- and S/G2/M-phase, we performed several physical and chemical assays. Results indicated that: 1) the membranes of cells in G1-phase contained greater amounts of sialic acids (glycoproteins) than the membranes of cells in S/G2/M-phase; 2) the membrane stiffness of cells in S/G2/M-phase is more rigid than those in G1-phase by measurement using optical tweezers; and 3) S/G2/M-phase cells contained higher content of Gb3, Gb4 and GlcCer than G1-phase cells by an assay for lipid composition.

**Conclusions:**

A novel single-virus infection system was developed to characterize the difference in influenza virus susceptibility between G1- and S/G2/M-phase cells. Differences in virus binding specificity were associated with alterations in the lipid composition, sialic acid content, and membrane stiffness. This single-virus infection system will be useful for studying the infection mechanisms of other viruses.

## Introduction

The influenza virus particle is spherical, about 100 nm in diameter, and encapsulated by a lipid membrane derived from the host cell. Two surface glycoproteins, hemagglutinin (HA) and neuraminidase (NA), encoded by the virus genome are localized to the viral envelope. HA binds specifically to sialic acids, which serve as receptors for virus attachment [1]. After binding to sialic acids on the host cell membrane, the virus particle enters into the cytoplasm by endocytosis [2], [3], [4]. Human influenza viruses preferentially bind to sialic acids containing α2-6 linkages [Neu5Ac(α2-6)Gal], whereas avian influenza viruses show a preference for α2-3 linkages [5], [6], [7]. The influenza virus envelope fuses with the endosomal membrane via HA during trafficking towards the perinuclear region [8]. The genome is then released and transported to the nucleus, where replication and transcription take place. Influenza virus RNA-dependent RNA polymerase (RdRp) synthesizes two different RNA species (mRNA and cRNA) from a single template (vRNA). Capped host-cell RNAs are required for viral mRNA synthesis as a primer by influenza virus RdRp [9], and thus the growth of influenza virus correlates the level of capped RNA in the cell. Along this line, it is noteworthy that the level of cellular mRNA synthesis is higher in G1- than in S/G2/M-phase cells [10]. We then hypothesized that influenza virus infection occurs at a certain phase of the cell cycle with higher level of mRNA production.

Influenza virus RdRp composed of three virus-coded subunits, PB1, PB2 and PA, and the RdRp in viral particle catalyzes transcription [11], but in virus-infected cells, the influenza virus RdRp catalyzes both transcription and replication by conversion from transcriptase to replicase by a host factor(s). Thus, besides the level of host cell mRNA, the growth of influenza virus depends on the putative host factor(s), such as factor(s) involved in conversion of the RdRp. Previously we screened for host factors interacting with influenza virus RdRp. One of these is ErbB3 binding protein 1 (Ebp1), which interacts with the PB1 subunit of influenza viral RdRp and interferes with its function [12]. Ebp1 plays various roles in cell growth and differentiation [13]–[18]. Ebp1 is expressed in cell cycle-dependent manner, being expressed in G1- and S-phase [19]. These observations altogether indicate cell cycle-coupled changes in influenza virus susceptibility. Up to the present, however, no direct determination of influenza virus susceptibility was performed between cells with different phases.

The nuclear membrane is disassembled during S/G2/M-phase, prior to cell division, and subsequently reassembled after cell separation. Furthermore, the cell shape alters dynamically during the cell cycle [20]. These changes imply that the composition of cell membrane alters during the cell cycle; however, the differences in membrane structure between G1- and S/G2/M-phase are currently unknown. The phase, in which influenza virus attaches to cells, has not been identified, but recently, He *et al*. [21] demonstrated that influenza virus replication induces cell cycle arrest in G0/G1-phase. We, therefore, tried to identify whether the influenza virus preferentially binds to host cells at specific phases of the cell cycle. For this purpose, virus infection using a synchronized cell could be a choice, but synchronization was difficult for H292 cells. To determine which phase of the cell cycle is most susceptible for influenza virus attachment, we then developed a single virus infection system using optical tweezers [22]. Previously optical tweezers were used to examine interactions between biological molecules attached to microspheres. Case *et al*. [23] used optical tweezers to demonstrate how condensin induces DNA compaction while Wang *et al*. [24] stretched DNA using optical tweezers. Chen *et al*. [25] studied the interaction between a natural killer cell and anti-Lymphocyte associated with antigen-1(LFA-1) using optical tweezers, with results indicating that anti-LFA-1 antibody may cause clustering of LFA-1 on the NK cell surface. These results indicate that optical tweezers are an attractive tool to characterize the force of protein-protein interactions.

In this study, using optical tweezers we show that the influenza virus attaches preferentially to cells in the G1-phase rather than those in the S/G2/M- phase. To our knowledge, this is the first successful application of optical tweezers for virus infection into individual cell.

In addition, our results indicate that the sialic acid content of the membrane was higher on cells in G1- than in S/G2/M-phase. In concert the difference in sialic acid content of the membrane, we also succeeded to measure the difference in membrane stiffness between G1- and S/G2/M-phase cells using optical tweezers. The chemical and physical changes in membrane were associated with high-level expression of globotriaosylceramide (Gb3), globotetraosylceramide (Gb4), and glycosylceramide (GlcCer) in S/G2/M- than G1-phase cells. Taken together we propose a molecular basis of the cell membrane influencing influenza virus susceptibility.

## Results

### Preferential binding of influenza virus to G1-phase cells

**Figure 1:**
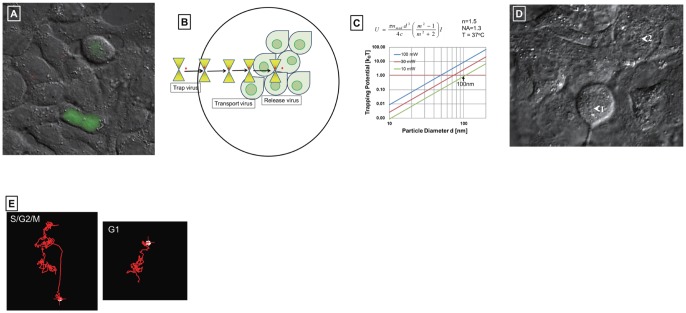
Attachment of DiI-labeled influenza virus particles to cells at different phases of the cell cycle. A. H292 cells were transfected with pFucci-S/G2/M Green vector and cultured overnight. GFP expression was observed only in S/G2/M-phase. DiI-labeled virus particles were added to cultured cells and incubated for 15 min at 34°C. Unbound virus was washed off with PBS and the cells were fixed with 4% paraformaldehyde and observed under a Nikon Ti E confocal microscope fitted with a 100× objective lens. The red particles are DiI-labeled viruses. The green colored cells express GFP. B. Cartoon of virus trapping and release on a cell using optical tweezers. Yellow triangle represents optical tweezers; red circle represents virus; light green colored teardrop shape represents the cell. C. Trapping potential was calculated under the indicated conditions. n, refractive index; NA, numerical aperture; T, absolute temperature; blue, red, and green lines represent laser power is at 100 mW, 30 mW, and 10 mW, respectively. D. The cells in the microchip were observed through the objective lens (×100). The DiI-labeled virus is trapped in the chamber of the microchip and transported to the apical membrane of a mitotic cell (arrow 1) but was unable to attach. The same particle was recaptured and then transported to the apical membrane of a G1-phase cell (arrow 2). E. Trace showing virus particle movement after transportation to a dividing (left) or resting (right) cell. White circle (red-cross) in the left panel represents the recaptured virus, whereas that in the right panel represents that the brownian motion of the virus particle on the cell membrane has stopped.

To determine whether influenza virus binds preferentially to cells at a particular phase of the cell cycle, purified influenza virus particles were labeled with a fluorescent probe, 1,1′-dioctadecyl-3,3,3′,3′-tetramethylindocarbocyanine perchlorate (DiI). First, the binding of the fluorescence-labeled virus to host cells was confirmed by HA titration using chicken erythrocytes (see Materials and Methods). The DiI-labeled virus showed only a small difference in infectivity, a less than 2-fold reduction in HA titer compared to unlabeled virus (data not shown). Binding of DiI-labeled virus to the sialidase treated cell membrane was examined. For this purpose we performed the following two experiments, 1. binding to the sialidase treated H292 cells (human lung mucoepidermoid carcinoma cells), 2. aggregation of the sialidase treated chicken erythrocyte. The DiI-labeled virus particles were added to semi-confluent H292 cells. After incubation for 15 min, the unbound virus particles were removed. Then the cells were fixed with paraformaldehyde and examined by oil immersed microscopy (100× magnification). The binding ratio of the DiI-labeled virus on sialidase treated cells was 0.5% (one DiI-labeled virus bound cell in 200 cells), while the binding ratio of that on sialidase untreated cells was 7% (14 DiI-labeled virus bound cells in 200 cells). Namely, the DiI-labeled virus did not bind on sialidase treated cell membrane (Fig. S1A) and the sialidase treated chicken erythrocyte did not aggregate with the DiI-labeled virus as same as the DiI-unlabeled virus (Fig. S1B). These results indicate that the receptor binding mechanism of the fluorescence-labeled virus was as same as that of the fluorescence-unlabeled virus. To elucidate the binding specificity of influenza virus, H292 cells were transfected with pFucci-S/G2/M Green vector (a fluorescent ubiquitination-based cell cycle indicator, MBL) prior to the addition of DiI-labeled influenza virus. To estimate the transfection efficiency, pEGFP-C1 vector (Clontech) was transfected into H292 cells as a transfection standard. The transfection efficiency was estimated to be >90%. In order to elucidate the binding specificity of H292 cell in different phases, DiI-labeled influenza virus particles were added to semi-confluent H292 cells transfected with pFucci-S/G2/M Green vector (at moi 1). After incubation for 15 min, unbound viruses removed and then cells were fixed and examined by oil immersion microscopy (100× magnification). The microscopic observations indicated that the attachment of influenza virus particles to H292 cells was not uniform, suggesting the cell cycle-dependent variation in virus susceptibility (Fig. 1A and S2).

Influenza virus attached cells were easily detected by the fluorescent probe Dil, while cells in S/G2/M-phase were detected by the GFP expressed from pFucci-S/G2/M Green vector. Among 50 Dil-labaled influenza virus attached cells that the pFucci-S/G2/M Green vector were transfected, 48 cells were not observed GFP expression, but only 2 cells were observed GFP expression. It was examined whether the different fluorescence-labeled influenza virus could attach to the specific cell phase. For this purpose the influenza virus particles were labeled with DiO or Syto21 respectively. DiO intercalates into lipid bilayer, whereas Syto21 binds to RNA. As shown in Fig. S2, the different fluorescence-labeled influenza viruses bound on G1-phase cells as same as DiI-labeled virus. These results indicate that fluorescence-labeled influenza virus did not bind non-specifically on the cell, namely, influenza virus preferentially bound onto G1-phase cells.

### Single-virus trapping by optical tweezers and infection of G1-phase cells

Previously, we developed a thermo-reversible hydrogel and optical tweezers to infect a cell with DiI-labeled influenza virus in a microchip [Fig. 1B and ref. 22]. Because of it was thought that the influenza virus particle was too small to be manipulated using optical tweezers. Using this system, we succeeded in trapping DiI-labeled influenza virus covered with gelated thermo-reversible hydrogel by using a laser, and infection of Dil-labeled influenza virus into a single cell. Infection was confirmed by detection of the PB1 subunit of influenza virus RdRp by immunostaining of the cell using anti-PB1 serum at 4 h after virus attachment [22].

In this study, however, we modified this system to eliminate the need for the thermo-reversible hydrogel and used a new technique to evaluate the binding of DiI-labeled influenza virus to individual G1- or S/G2/M- phase cells. Before trapping of DiI-labeled influenza virus in the absence of thermo-reversible hydrogel, we first examined new system whether it could be possible to trap a 100 nm particle in diameter using a 1064 nm laser. The feasibility of trapping small particles is determined by the relationship between trapping potential, exerted by the radiation-pressure force of the laser beam, and the thermal energy of the particle (k_B_ *T*; where k_B_ is the Boltzmann constant and *T* is the absolute temperature). If the particle is sufficiently smaller than the wavelength of light, scattering phenomena can be described as Rayleigh scattering, in which the dominant component of the radiation force on the particle is the gradient force, exerted by the gradient of light intensity. The radiation force in Rayleigh scattering is proportional to the polarizability of a particle and the third power of particle radius [26]. The trapping potential *U*, is given as *U = *π*n_0_ d* ^3^ *I* ((*m* ^2^−1)/(*m* ^2^+2))/(4c), where *I* is the irradiance of light, *n_0_* is the refractive index of the surrounding medium, *m* is the relative refractive index of a particle against the surrounding medium, and *d* and c represent the particle diameter and light velocity, respectively [26]. By using this equation, the trapping potential in our experiment can be calculated as 2.2 k_B_ *T*, by assuming as a laser intensity of 25 mW, and a particle refractive index and diameter of 1.5 and 100 nm, respectively.

Consequently the particle can be trapped in a focused laser beam (Fig. 1C). Since the influenza virus consists of an RNA genome and proteins (HA, NA, M1, M2, NEP, NP, RdRp), in addition to some cellular proteins and lipid from the host cell, and has a diameter of approximately 100 nm, it is possible to trap the virus using optical tweezers. Theoretically, from the above equation, the laser power required to trap an influenza virus particle is 100 mW. Experimentally, we used a laser power of 600 mW in a microchip with a cell chamber connected by a tunnel (50 μm^2^×500 µm) to a virus pool to trap a single influenza virus by optical tweezers (Fig. 1C). The DiI-labeled virus was trapped from the virus pool and transported to the cell chamber (Fig. 1B). Figure 1D shows cells grown in the chamber that are ready for infection with DiI-labeled virus using optical tweezers. When the DiI-labeled virus was released on S/G2/M- phase cell (cell 1), the virus particle moved quickly but did not bind to the S/G2/M-phase cell. Then the unbound virus was recaptured, transported, and released on a G1-phase cell (cell 2), the virus quickly bound to the G1 phase cell (Fig. E, also see Movie S1) and the bound virus could not be recaptured. The single-virus infection was repeated more than ten times, and observed the preferential binding on to G1 phase cells.

### Confirmation of single-virus infection using optical tweezers

To confirm the influenza virus infection by using optical tweezers, the DiI-labeled virus-bound cell was subjected to RT-PCR assay for detection of influenza virus transcripts. At 6 h after the virus attachment, both the virus-bound and -unbound cells were removed by suction separately (Fig. S3) and RT-PCR was performed for detection of influenza virus PB1 and PB2 mRNAs, with 18 S rRNA as a control. As shown in Table 1, both PB1 and PB2 mRNAs were detected in the cell bound by the viral particle but not in the viral particle unbound cell. These results altogether indicate that influenza virus binding is selective for G1-phase cells. Interestingly, the manipulation of the DiI-labeled virus using optical tweezers without the thermo-reversible hydrogel allowed us to observe brownian movement of the virus on the cell (Fig. 1E and Movie S1). Figure 1E shows traces of the movement of the virus after release on cells in either G1- or S/G2/M-phases. The different movement patterns of the virus on G1- and S/G2/M-phase cells suggest that receptor content may be different between them.

**Table 1:** PB1 and PB2 mRNA in single-virus infected cells.

	Ct	
RNA	Uninfected	Infected
18S rRNA	33.4 (0.02)	33 (0.01)
PB1 mRNA	ND	19.3 (0.02)
PB2 mRNA	ND	20 (0.02)

Detection of PB1, PB2 mRNA in single virus infected cells by RT-PCR. Individual cells, either infected with DiI-labeled influenza virus using optical tweezers or uninfected (n = 5 for each cells, virus bound and unbound cells), were removed by suction at 6 hpi and assayed for mRNA of PB1 and PB2, and 18S rRNA. The numbers in parentheses indicate standard deviation from n = 5.

### Comparison of the sialic acid content between G1- and S/G2/M-phase cells

As an attempt to understand the molecular basis of the influenza virus susceptibility between G1- and S/G2/M-phase cells, we examined the difference in cell surface structure. Since influenza virus binds to the sialic acid on cell membranes, we examined the sialic acid content of cell membranes. Prior to analysis of the sialic acid content, a solid-phase virus binding assay [27] was carried out to identify the form of sialic acid recognized by the influenza virus in this experiment. The result indicated that the influenza virus used here preferentially attached to α2-3-linked sialic acid residues (data not shown). Live H292 cells were then stained with Vybrant DyeCycle Stain and sorted by flow cytometry. The sorted cells were combined and stored in −80°C freezer until use. An aliquot of sorted cells was taken to measure the total protein content in each cell phase, and which was very similar (data not shown).

**Figure 2:**
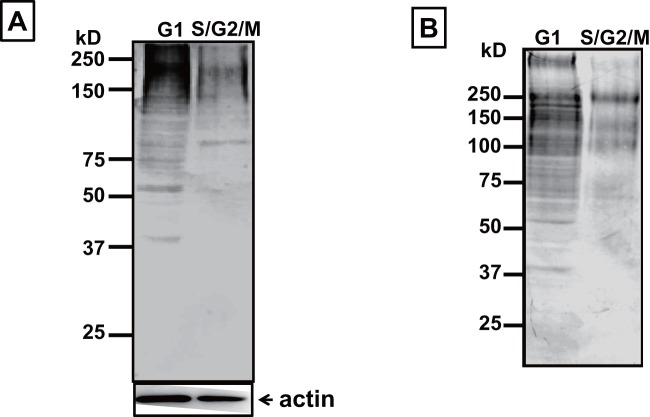
Comparison of sialic acid between cells in G1- and S/G2/M- phase. H292 cells were stained with Vybrant DyeCycle Stain, and G1- and S/G2/M-phase cells were separated using a cell sorter (FACSCalibur). The sorted cells were lysed in extraction buffer followed by SDS-PAGE and blotting on PVDF membrane. A. Sialic acid with α2-3 linkage was detected with digoxigenin-labeled lectin (Maackia amurensis) and an anti-digoxigenin antibody conjugated with alkaline phosphatase. B. Sialic acid with α2-6 linkage was detected with digoxigenin-labeled lectin (Sambucus nigra) and an anti-digoxigenin antibody conjugated with alkaline phosphatase.

After the cell lysate was separated on 8% SDS-PAGE and transferred to a PVDF membrane (see Materials and Methods), the levels of sialic acids were determined using digoxigenin-labeled lectins [6]. Interestingly, the level of α2-3-linked sialic acid in G1-phase cells was greater than in S/G2/M-phase cells (Fig. 2A), and the result of α2-6-linked sialic acid was same as α2-3-linked sialic acid (Fig. 2B). These results indicate that influenza virus binds preferentially to G1-phase cells with higher level of sialic acid content.

### Comparison of membrane stiffness between G1- and S/G2/M-phase cells

**Figure 3:**
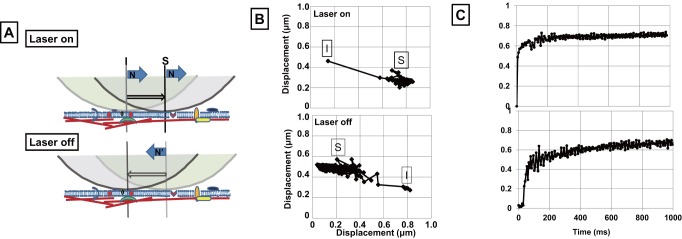
Measurement of membrane stiffness using optical tweezers . A. Diagram of the displacement of the bead by the optical tweezers. A collagen-coated bead (2 μm in diameter) was attached to the cell membrane using optical tweezers and held in position for a short time. ‘Laser on’, the path of the bead forced by the laser; ‘laser off’, the path of the bead without laser force. Blue arrow, forced direction of the bead; arrow outlined in black, displacement of the bead. Colored forms, membrane proteins; red bars, structural proteins; hemispheres, beads; I, initial position of the bead; S, stopping position of the bead; B, tracing of the bead with the laser on or off; C, tracing of the displacement of the bead for 1000 ms.

Since the glycoprotein content of cell membranes differs between G1- and S/G2/M-phase cells (Fig. 2), we next analyzed the membrane stiffness for these cells. For estimation of the membrane stiffness, we developed a system to measure membrane stiffness using optical tweezers. In this assay, a collagen-coated bead (2 μm in diameter) was adhered to integrin molecules by holding it against the cell membrane for thirty seconds using optical tweezers (1064 nm) [28]. Then the optical tweezers (1064 nm) at 0.6 W was directed at the bead, and began to move it along the cellular membrane. When the bead stopped moving, the laser power was turned off (Fig. 3A). The traced path of a bead is shown in Figures 3B and 3C. The bead began to move rapidly once the laser was turned on, and then settled in a certain position and remained there as long as the laser remained on (Figs. 3B and 3C; laser on). After the laser was turned off, the bead returned to its original position but remained in motion for 100 m sec (Figs. 3B and 3C; laser off). This method can be used to measure membrane stiffness, which correlates with the displacement of the bead. We initially tried to measure the movement of the bead under the laser at several sites on the membrane. Stable movement was only observed at the region near the nuclear membrane and the endoplasmic reticulum (data not shown); therefore, further assays of cellular membrane stiffness were carried out at this site.

**Figure 4:**
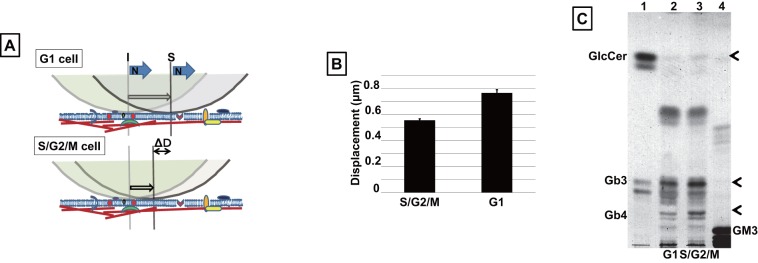
Comparison of membrane stiffness and lipid composition between cells in G1- and S/G2/M-phase. A. Diagram of the measurement of membrane stiffness using optical tweezers. A collagen-coated bead (2 μm in diameter) was attached to the cell membrane using optical tweezers and held in position for 30 sec. The trapped bead was then forced along the membrane using the optical tweezers. N, force applied; ΔD, the difference in the displacement of the bead between cells in G1- and S/G2/M-phase. B. Comparison of the membrane stiffness between cells in G1- and S/G2/M- phase. The signal generated by the movement of the particle was then calculated. C. Cells were sorted into G1- and S/G2/M-population. The lipids were extracted with chloroform:methanol, separated on a TLC plate, and visualized with orcinol. lanes 1 and 4, lipid standard marker; lane 2, G1-phase; lane 3, S/G2/M-phase.

Membrane stiffness of G1- and S/G2/M-phase cells was then measured using this method. Figure. 4A shows an example of the movement of a bead on the membrane. Fifty cells in each of G1- and S/G2/M-phase were measured using this technique. Interestingly, the displacement of the bead on S/G2/M-phase cells was shorter than on G1-phase cells, indicating increased membrane stiffness in the S/G2/M-phase of the cell cycle (Fig. 4B). The reason for the increased stiffness of S/G2/M-phase cell membranes was unclear; therefore, to understand the underlying mechanism, further assays were carried out to characterize membrane composition.

### Identification of the lipid composition for G1- and S/G2/M-phase cells

To understand the molecular basis of the difference in membrane stiffness between G1- and S/G2/M-phase cells, we next analyzed the lipid composition of cell membrane. Cells were sorted into G1- and S/G2/M-phase cells and lipids were extracted from 3×10^6^ cells of each cell sample using chloroform:methanol (2∶1 v/v), and then separated by thin layer chromatography (TLC) in chloroform:methanol:water (40∶12∶1 v/v/v) and visualized by spraying the plate with orcinol in 70% sulfuric acid. As shown in Fig. 4C (lanes 2 and 3), the expression of Gb3, Gb4, and GlcCer was higher in S/G2/M-phase cells than in G1-phase cells. To understand the relationship between the lipid content and the membrane stiffness between G1- and S/G2/M-phase cells, further studies are required.

## Discussion

The optical tweezers methods have been developed recently and used for discovering interactions between biological molecules such as DNA-protein. In addition, most of these methods used molecules adhered to microbead to provide force for the manipulation of biological molecules. For manipulation of biological molecules using optical tweezers, the biological molecules are often bound to microbeads. Using homogenous preparation of silver nanoparticles, the bead size capable of being trapped by optical tweezers (1064 nm) was reported to be in the range 20–275 nm [29]. In agreement with this report, we demonstrated in this study the successful trapping of influenza virus of the size of approximately 100 nm, however, the two studies used nanoparticles differing in composition, as those used in the previous study were homogeneous (silver), whereas those in this study were heterogeneous (protein, lipid and nucleic acid). Recently Sieben *et al*. [30] determined the force of the interaction between HA and sialic acid on the cell membrane using optical tweezers and an HA-coated bead. By contrast, we developed a novel method, using optical tweezers to trap heterogeneous virus particles. The laser power of optical tweezers used for this trapping was 0.6 W, and this power is enough to trap virus particle, because a laser power of 0.1 W is enough for trapping a 100 nm particle as estimated from the trapping potential equation and a graph (Fig. 1C).

Most viruses bound host cells enter through receptor-mediated endocytosis, but the entry mechanisms remain poorly understood. Initial attempts have been made to follow these processes using improved methods such as fluorescent probe for labeling virus and real-time imaging. For instance, Cureton *et al*. [31] showed that Vesicular Stomatitis Virus induced the nucleation of clathrin to mediate its uptake. By tracking of fluorescent-labeled virus particles, Rust *et al*. [32] demonstrated that influenza virus entered into host cells through various pathways including both clathrin-mediated and clathrin-independent endocytic pathways. Ewers *et al*. [33] overviewed the strategies for fluorescence labeling of viruses and for tracking single virus particle into host cells. In this study, we developed a novel single virus infection system using optical tweezers to understand the specificity of influenza virus binding to the cell membrane. To the best of our knowledge, this is the first demonstration of trapping of fluorescence-labeled influenza virus (about 100 nm in diameter) using optical tweezers. Using the successfully trapped virus particle, we also showed that influenza virus binds selectively to the membranes of G1-phase cells. The selective binding of influenza virus to G1-phase cells encouraged us to study the molecular basis of differences in membrane composition between G1- and S/G2/M-phase cells. Previously, Guo *et al.* [27] showed that once sialic acids are removed by sialidase, influenza virus is unable to bind to the membrane. Our experiment also indicated that the fluorescence-labeled influenza virus did not bind on sialidase treated cells. After analysis of membrane composition, we found that the level of sialic acid of α2-3-linkage is higher in the membranes of G1-phase cells than in S/G2/M-phase cells. Sialic acid with α2-3-linkage was detected by using digoxigenin-conjugated lectin following SDS-PAGE separation of cell lysates; this method allowed the detection of α2-3-linked sialic acid residue bound to protein. We are currently screening such proteins with α2-3-linked sialic acid residue, which could be the binding target of influenza virus. Along this line, it is noteworthy that the high level of sialic acids with α2-3-linkage in G1-phase cells is also of interest as this finding indicates that some glycoproteins may play a critical role in cell cycle progression from S/G2/M- to G1- phase. The major differences between G1- and S/G2/M-phases are nuclear division, chromosome condensation, and disappearance of the nuclear membrane; however, differences in both physical conditions and chemical composition between G1- and S/G2/M-phase cells are not yet fully elucidated. In this study, we show that cells in S/G2/M-phase have increased membrane stiffness compared to those in G1-phase, although the components regulating this difference in membrane stiffness remain unclear. To understand the mechanisms controlling membrane stiffness, it will be necessary to assay the protein composition of cell membranes in both S/G2/M- and G1- phase, because the observed differences in Gb3, Gb4 and GlcCer content may be insufficient to explain the differences in membrane stiffness. Further studies are required to understand the role of Gb3, Gb4 and GlcCer in the membrane.

**Figure 5:**
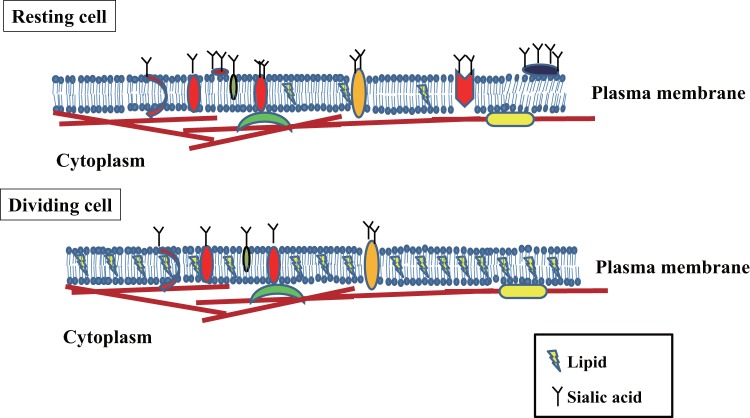
Summary of results. Lipid content was higher in cells in S/G2/M- phase than G1-phase; however, sialic acid content was higher in G1-phase cells.

In summary, the newly developed single-virus infection system allowed to identify the biological difference between G1- and S/G2/M-phase cells in influenza virus attachment, and to highlight physical and chemical differences between these cells. The difference of amount of both sialic acid and lipid between G1- and S/G2/M-phase cells is diagramed in Fig. 5. However, further investigation will be required to identify the molecular basis including the trigger for sialic acid synthesis after cell division and to resolve questions regarding the relationship between membrane stiffness and lipid/protein composition.

## Materials and Methods

### Cell lines

H292 cells (human lung mucoepidermoid carcinoma cells) purchased from ATCC were cultured in Minimum Essential Medium (MEM) supplemented with 10% fetal calf serum under 5% CO_2_ atmosphere at 37°C.

### Virus growth and fluorescence labeling

Influenza virus A/PR/8/34 (wild type) was propagated in 10-day-old embryonated chicken eggs and the virus titer was determined by measuring HA activity and plaque assay. HA titration was carried out using chicken erythrocytes (a gift from Dr. Iwata, Nippon Institute of Biological Science, Ome, Tokyo). The virus solution was diluted stepwisely with PBS in round–bottomed 96-well plate, and added to 0.5% chicken erythrocytes in PBS. After mixing, the plate was placed at room temperature for 1 h and then inspected the aggregation of red blood cells. Before labeling the virus particles, DiI and DiO (Invitrogen/Life Technologies, USA) were dissolved in dimethyl sulfoxide (DMSO) and diluted with PBS to a concentration of 2 μg/ml. Influenza virus in allantoic fluid was incubated with the DiI, DiO and 0.5 mM Syto21 (Invitrogen/Life Technologies, USA) solution separately for 30 min at room temperature. The virus in fluorescence solution was applied to a spin column containing Sephadex G50 beads (Pharmacia, USA) and centrifuged for 2 min at 3000 rpm to remove excess fluorescence. The flow-through fraction was used for manipulation of single virus.

### Sialidase treatment of H292 cells and chicken erythrocytes

8.5×10^5^ chicken erythrocytes and 10^6^ cells of H292 adhered on glass bottom dish were incubated with 30 U of α2-3-sialidase derived from Arthrobactor ureafaciens for 1 h at 37°C with gentle rocking respectively. After incubation, the cells were washed with PBS by centrifugation at 2000 rpm at 4°C for three times.

### Microscopic observation of fluorescence-labeled influenza viruses on cells

Fluorescence-labeled influenza viruses were added to semi-confluent cells at moi 1 and incubated for 15 min at 34°C. After removal of unattached influenza virus particles, cells were washed twice with PBS and fixed with 4% paraformaldehyde for 15 min at room temperature. Fluorescence-labeled influenza viruses attached to the cell membrane were observed using a Nikon TiE microscope fitted with a 100× objective lens. To distinguish S/G2/M- from G1-phase cells, H292 cells were transfected with the pFucci-S/G2/M Green vector (MBL,USA) by electroporation at 1200 V using a MicroPorator (Seoul, Korea) and cultured overnight at 37°C in 5% CO_2_. Fluorescence-labeled virus was then added to the pFucci-S/G2/M Green transfected cells.

### Manufacture of the microchip for single virus infection

A microchip comprising two chambers on a glass coverslip (Matsunami Glass, Japan) was manufactured from the silicon elastomer polydimethylsiloxane (PDMS; Dow Corning Toray Silicone, USA). One chamber was used for cell culture and the other for the virus pool. The two chambers were connected by a narrow tunnel of 50×50 μm^2^ in square and 500 μm in length. A tube was used to supply 5% CO_2_ to the cell culture chamber. The total area of the chamber available for cell culture was approximately 21 mm^2^. The glass coverslip was coated with lysine to allow cell attachment.

### Manipulation of the DiI-labeled virus using optical tweezers

DiI-labeled influenza virus particles were injected into the virus chamber of the microchip, trapped using a 1064 nm laser oscillator (maximum power 2.0 W; Sigma Koki, Tokyo, Japan) viewed through a 100× oil immersion objective lens mounted on an inverted microscope (Nikon Eclipse Ti; Nikon Instruments, Tokyo, Japan), and then transported through the tunnel to cell and released onto cells.

### Immunostaining of influenza virus bound cells

The cells of influenza virus attached and unattached cells were assayed by immunostaining using anti-PB1. At 6 h after virus attachment on the cell, the cells were washed with PBS and fixed with 4% paraformaldehyde solution, treated with 0.5% Triton X-100, blocked with 1% BSA. After blocking, the cells were incubated with anti PB1 antiserum for 1 h, washed with 1% BSA/PBS solution and then incubated with anti-rabbit IgG with Alexa 488 for 1 h at 37°C and observed under the objective lens (×100).

### Single-cell manipulation and RT-PCR

The cell infected with fluorescence-labeled virus in the culture dish was manipulated with a glass capillary using Pico-Pipet (Altair, Japan). Before suction of the single cell, the cultured chamber was washed with 0.04% EDTA in PBS twice and incubated in the same solution at room temperature for 10–20 minutes. Then, using the capillary, the focused cell was transferred to a 1.5 ml tube. Using an RT-PCR kit (Qiagen, Germany), mRNA was extracted and RT-PCR was performed (AB 7500 Real Time PCR System). Reaction products were detected using SYBR green. The used primers for RT-PCR were shown in Table 2.

**Table 2:** Primers used for RT-PCR.

segment	primer sequence
PB1(forward)	5′-TGAGGAATCCCATCCTGGTA-3′
PB1(reverse)	5′-ATAGGTCTGTCGGCCTTGTG-3′
PB2(forward)	5′-TGATGGTTGCATACATGTTGGA-3′
PB2(reverse)	5′-CAGCCACTGGGAGGAATCTC-3′
18SrRNA(forward)	5′-ACTCAACACGGGAAACCTCA-3′
18SrRNA(reverse	5′-AACCAGACAAATCGCTCCAC-3′

The primers used for RT-PCR were synthesized by Takara (Shiga, Japan).

### Sorting of G1- and S/G2/M-phase cells

Cultured cells (10^7^) were stained with Vybrant DyeCycle Stain (Molecular Probes/Life Technologies, USA) for 60 min at 37°C and detached from the dish using a solution of 0.04% EDTA in PBS. The cell suspension was then sorted using a FACSCalibur flow cytometer (Becton Dickinson, USA). Sorted cells were combined and stored at −80°C until used for a further assay (e.g., for sialic acid content, membrane lipid assay). The protein content of 10^4^ cells from each phase was measured using a Bio Rad Kit (Bio Rad, USA).

### Sialic acid assay

G1- and S/G2/M-phase cells (10^6^ of each) were collected after flow sorting, lysed in 100 μl of hypotonic buffer [10 mM HEPES (pH 7.8), 10 mM KCl, 1.5 mM MgCl_2_, 2 mM DTT, 0.1% Triton X-100, and 1 mM PMSF] using glass beads, and subjected to centrifugation at 700×*g* for 10 min in a TOMY MX-301 centrifuge. The pelleted fraction was then treated with 100 μl of extraction buffer [20 mM HEPES (pH 7.8), 1.5 mM MgCl_2_, 0.2 mM EDTA, 0.5 M NaCl, 25% glycerol, 1 mM DTT, and 0.5 mM PMSF] and centrifuged at 186,000×*g* in a Beckman Optima ultracentrifuge (Beckman Coulter, USA). Ten microliters of the supernatant clarified by further centrifugation at 186,000×*g* was subjected to SDS-PAGE and transferred onto a PVDF membrane. To detect sialic acid, the membrane was probed with digoxigenin-conjugated lectins, which was then detected with an alkaline phosphatase-conjugated anti-digoxigenin antibody (Roche Molecular Diagnostics, USA).

### Identification of the receptor specificity of influenza virus HA

Receptor specificity was determined using a solid-phase binding assay as described previously by Guo et al [27]. Two glycopolymers were added to a Universal-Bind Costar microplate (Cole Palmer, USA), followed by UV irradiation (254 nm) for 10 min. Excess glycopolymer was removed and the plate washed with PBS. The influenza virus solution was then added and incubated for 1 h at 4°C. After incubation, the wells were washed three times with PBST (3.2 mM Na_2_HPO_4_, 0.5 mM KH_2_PO_4_, 1.3 mM KCl, and 0.05% Tween 20; pH 7.4), and fixed with 10% formalin in PBST for 30 min. The binding of virus particles to glycopolymers was detected by incubation with an antibody against influenza virus A/PR/8/34 for 60 min at 37°C.

### Assay of lipid composition using TLC

Cellular lipids were extracted from 3×10^6^ cells in 20 volumes of 2∶1 chloroform:methanol. The extracts were separated by TLC in chloroform:methanol:water (40∶12∶1 v/v/v) and visualized by spraying with orcinol in 70% sulfuric acid.

### Assay of membrane stiffness using optical tweezers

Type I collagen was coated onto carboxylate particles (2 μm in diameter) (Spherotech Inc., USA) via the cross-linker 1-Ethyl-3- (3-dimethylaminopropyl) carbodiimide hydrochloride (EDC), using the method of Suzuki [34]. A collagen-coated particle was then adhered to the surface of a cell with optical tweezers and forced to move once, laterally, through an amplitude of 810 nm (half width), also using optical tweezers. The movement was observed under a microscope. After processing the resulting images, the displacement of the bead from the initial position was calculated.

## Supporting Information

Figure S1 **Observation of binding activity of DiI-labeled virus on the cell treated with sialidase.** A. DiI-labeled influenza virus was added to sialidase-treated (a) and –untreated (b) H292 cells respectively, and incubated for 15 min. The unbound viruses were removed and cells were washed with PBS and then fixed with 4% paraformaldehyde for 15 min at room temperature and then observed under a Nikon Ti E confocal microscope fitted with a 100× objective lens. Red colored particles indicated by arrow head in white represent virus particle. B. Chicken erythrocyte aggregation by DiI-labeled influenza virus was assayed. Fifty μl of DiI-labeled influenza virus was added in the 50 μl chicken sialidase-treated and -untreated erythrocyte each. After 1h incubation at room temperature, the aggregation of chicken erythrocyte was observed. wells 1, 2, 3 and 4, influenza virus (+); well 5, influenza virus (−); wells 1 and 2, DiI-unlabeled virus; wells 3 and 4, DiI-labeled virus; wells 1 and 3, sialidase-untreated chicken erythrocyte; wells 2 and 4, sialidase-treated chicken erythrocyte.
(EPS)

Figure S2 **Observation of various fluorescence-labeled influenza viruses on cell membrane.** The various fluorescence-labeled influenza viruses were added on the H292 cells that were transfected with pFucci-S/G2/M Green vector and incubated for 15 min. The unbound influenza viruses were removed and cells were washed with PBS, fixed with 4% paraformaldehyde for 15 min at room temperature and then microscopic observation was carried out under the ×100 objective lens. A. DiI-labeled influenza virus binding onto H292 cells. B. DiO-labeled influenza virus binding onto H292 cells, C. Syto21-labeled influenza virus binding onto H292 cells. Red colored particles indicated by arrow head in white represent virus particle, green color represents the expressed GFP.
(EPS)

Figure S3 **Suction of a single cell using a capillary.** Cells in the chamber were washed with 0.04% EDTA in PBS and suctioned with a glass capillary. The cell indicated by the arrowhead was manipulated. Left, before suction; right, after suction; below, manipulated cell.
(EPS)

Movie S1 **Real-time observation of influenza virus on the cell.** Movie showing the movement of a DiI-labeled influenza virus particle on an H292 cell following manipulation with optical tweezers.
(MOV)
